# Patterns of Clinical Reactivity in a Danish Cohort of Tree Nut Allergic Children, Adolescents, and Young Adults

**DOI:** 10.3389/falgy.2022.824660

**Published:** 2022-03-28

**Authors:** Nanna Juel-Berg, Lau Fabricius Larsen, Niels Küchen, Ida Norgil, Kirsten Skamstrup Hansen, Lars K. Poulsen

**Affiliations:** ^1^Allergy Clinic, Department of Dermatology and Allergy, Copenhagen University Hospital, Gentofte, Denmark; ^2^Department of Pediatrics, Copenhagen University Hospital, Herlev, Denmark

**Keywords:** allergic symptoms, clinical reactivity, oral food challenge, tree nut allergy, peanut allergy, sensitization, eliciting dose, allergen-specific IgE

## Abstract

**Background:**

Tree nut allergy is associated with severe reactions and poly-sensitization to other nuts and peanuts often occurs. There are regional differences in sensitization profiles that result in differences in clinical presentation. Denmark is located in a birch pollen endemic area, which could influence the allergy patterns due to pollen cross-sensitization.

**Objective:**

This study aimed to investigate patterns of sensitization and clinical reactivity to tree nuts and peanuts and threshold levels for oral food challenges (OFCs) in a Danish cohort of tree nut allergic children, adolescents, and young adults.

**Methods:**

Forty tree nut allergic subjects were assessed for clinical reactivity to six nuts, i.e., hazelnut, walnut, pistachio, cashew, almond, and peanut, by OFCs or convincing medical history of an immediate allergic reaction or tolerance. Clinical presentation and allergen-specific immunoglobulin E (sIgE) levels together with eliciting dose and rescue medication in OFCs were furthermore assessed.

**Results:**

Allergy to two or more tree nuts was observed in most cases. Hazelnut-walnut dual allergy was common but not exclusively observed as concomitant allergies. Allergy to cashew was coincided in all but one of the assessed subjects with pistachio allergy. Half of all assessed subjects were allergic to peanuts. Oral symptoms followed by a skin reaction were the most common symptomatology that lead to OFC cessation and subjects often presented with symptoms from two or more organ systems. OFC threshold levels were within the same range, but cashew was distinguished from other nuts by causing allergic symptoms at the lowest dose. Clinical reactivity and the allergy patterns were to some extent reflected by sIgE levels and by correlations in sIgE between the nuts.

**Conclusions:**

In this Northern European cohort, subjects with clinically relevant tree nut allergy were generally allergic to two or more tree nuts and close to half of them also to peanuts. There were two distinct and independent allergic phenotypes; the majority of hazelnut allergic subjects were also allergic to walnut, and all but one subject with cashew allergy were dual allergic to pistachio. These findings are consistent with a strong sIgE correlation between hazelnut and walnut and a close to total sIgE correlation between cashew and pistachio.

## Introduction

Tree nuts are some of the most frequent triggers of allergy to plant foods (1–3). The worldwide prevalence of confirmed tree nut allergy ranges from 0 to 1.6% (4). Eating habits and pollen exposure vary by geographical region, and therefore, regional differences in the allergic phenotype can be expected (5–9). Cross-sensitization occurs because of structural similarities between the immunoglobulin E (IgE)-binding epitopes in different allergens (10, 11). In Northern Europe, a birch pollen allergy-endemic area, cross-sensitivity between allergens in birch pollen (Bet v 1) and hazelnut (Cor a 1) is common (12–14). Pollen-associated tree nut allergy is often confined to local symptoms in the oral cavity, and it rarely leads to severe clinical reactions (15, 16). In tree nut allergic individuals, which are also sensitized to tree or grass pollen, there is, however, no certainty about which allergen that is the primary sensitizer.

Serological cross-sensitization between various tree nuts and between tree nuts and other plant foods is common (17–19) but does not always predict clinical cross-reactivity (20). Sequence homology at the amino acid level does not necessarily predict a uniform structure in the allergens' IgE-binding epitopes (21). Cross-sensitization to taxonomically related nuts is common (22). A study from Finland (13) that investigates skin prick test sensitization profiles of edible nuts found that sensitization occurred in the clusters: hazelnut-almond-peanut, cashew-pistachio, and pecan-walnuts. In the present work, we will use the term co-sensitization to express that an individual is reacting to two different nut extracts, as it would necessitate an inhibition study to formally confirm cross-sensitization of tree nut or peanut allergens.

Clinically relevant allergy toward nuts is often present at an early age (23) and is rarely outgrown (8, 24), but the natural course of sensitization and clinical reactions toward multiple nuts cannot always be predicted (25). A study by Andorf et al. (26) that aimed at investigating common clinical patterns in multi food-allergic patients found a high coincidence of cashew and pistachio nut allergy and of walnut, pecan, and hazelnut allergy in the same individuals. Here, patients allergic to two or more food items with moderate to high allergen-specific IgE (sIgE) to the food item in question were offered food challenges but were not systematically assessed for clinical reactivity to all nuts. Another study by Elizur et al. mapped patterns of clinical reactivity between six tree nuts in an Israeli population (27). They found that most participants only reacted to one or two nuts and that allergy to walnut was most common followed by allergy to cashew nut in sensitized individuals. Lastly, a recent multicenter study by Brough et al. (9) that includes children from London, Geneva, and Valencia found an overall rate of coexistent peanut, tree nut, and sesame seed allergy in 61% of children, which upon study inclusion had at least one confirmed allergy to the foods in question. There are no recent comprehensive studies systematically investigating clinical patterns in a tree nut allergic Northern European population, where differences in food and environmental exposure are to be expected.

The scope of the present study was to investigate the pattern of clinical reactivity to five commonly ingested tree nuts among a Danish cohort of children, adolescents, and young adults. As tree nut allergic patients are often allergic to peanuts (28–30), we also wanted to investigate how peanut allergy correlates with a tree nut allergy in this tree nut allergic cohort. We established symptomatology and threshold levels for clinical reactivity by oral food challenges (OFCs) and further investigated how the level of sIgE correlated with the clinical reactivity.

## Materials and Methods

### Study Population and Design

This was an observational study of patterns of clinical reactivity to five tree nuts and peanuts in a Danish cohort of tree nut allergic patients. Subjects were recruited from the Department of Pediatrics and the Allergy Clinic, Copenhagen University Hospital (CUH), Gentofte and Herlev, Capital Region of Denmark.

Our focus was to map patterns of clinical reactivity in patients with a clinically relevant tree nut allergy. We recruited patients by the following criteria (Table 1).

**Table 1:** Inclusion and exclusion criteria.

**Inclusion criteria were as follows:**
Age 1–23 years,
Specific IgE > 0.7 kU/L to at least one tree nut, and
Medical history of an immediate allergic reaction to at least one tree nut
**Exclusion criteria were as follows**
Severe chronic or systemic medical condition or significant mental illness and
Treatment with medications that may impact challenge outcomes


We searched for eligible participants among outpatients and newly referred patients.

### Assessment of Serology and Clinical Reactivity

Medical history, physical examination, and sIgE (ImmunoCAP, Thermo Fisher Scientific, Uppsala, Sweden) against tree nuts, peanut and birch pollen were obtained from all participants at inclusion. The most recent sIgE results were used for statistical analysis and in figures. sIgE results that were below the cut-off limit of 0.35 kU/L were conservatively set to 0.34 in statistical calculations.

Oral food challenges were performed to establish whether patients were allergic or tolerant to the nut in question. A convincing medical history of recent tolerance (regular consumption) or of an immediate (<1 h) systemic allergic response upon ingesting the nut was also accepted. Medical history of an allergic reaction was only accepted if there had been unambiguous objective symptoms to the nut in question. We will only use the term allergic when the abovementioned criteria for allergy to a nut are met. Participants were offered OFCs with hazelnuts, walnuts, pistachio, cashew nuts, almonds, and peanuts (all unroasted whole nuts, dried at ambient temperature) if clinical reactivity was either uncertain or had not been established.

Oral food challenges regime:

Age 1–6 years: open OFCs
Age 7 years and up: primarily double-blind placebo-controlled food challenges (DBPCFCs)

Titrated OFCs were stopped at the appearance of objective symptoms (positive challenge outcome) (31) or after tolerating a cumulative dose of 10 g of the nut (negative challenge outcome). There was a minimum of 30-min interval between doses, and challenge doses could be repeated in case of ambiguous symptoms. For DBPCFCs, convincing subjective symptoms could be accepted as positive. The most recent results from food challenges conducted before the date of inclusion were included in the data.

In patients aged 7+ years, DBPCFCs could be replaced with open OFCs in cases where subjective symptoms were not to be expected, or if there was a high expectancy of severe reactivity from prior medical history. In instances where it was regarded unsafe to perform or if patients refused a challenge, and there was no medical history of either allergy or tolerance, data were regarded as missing. This was the case in a total of 27 out of 240 (11%) patient-food combinations.

### Assessment of Atopic Co-morbidity

Medical history, physical examination, and lung function measurements were used to assess patients for past or present asthma and allergic rhinitis. The UK Diagnostic Criteria for Atopic Dermatitis (32) were used to document past or present atopic dermatitis (AD).

### Ethical Approval

The study was approved by the local Danish Ethical committee, ID: H-6-2014-018. All participants (or their legal guardians) signed informed consent for participation.

### Statistical Analysis

The statistical software R version 3.2.4 (www.R-project.org/) was used for drawing distributions, scatterplots, and calculating Spearman correlations.

GraphPad Prism 7 (www.graphpad.com/scientific-software/prism/) was used for creating dose-response curves, performing Mann-Whitney U tests, unpaired *t*-tests, and Two-way ANOVA, multiple comparisons of IgE levels. Values of *p* < 0.05 were considered significant.

## Results

### Patient Characteristics

Forty subjects who were aged 2–23 were included in the study.

Mann-Whitney *U*-test yielded no significant differences in participant characteristics (allergen-specific IgE levels to tree nuts, peanut, and birch-pollen, gender, age, or ethnicity) between patients recruited *via* medical records (*n* = 21) and the newly referred patients (*n* = 19) (data not shown).

The median age at inclusion was 8 years of age, and 68% of study participants were of the male sex. All but one participant was known with prior or present atopic co-morbidity, where AD affected 88%, followed by asthma in 78% and rhinoconjunctivitis (RC) in 68%. The participant characteristics are summarized in Table 2.

**Table 2:** Participant's demographics, morbidity, and sensitization patterns.

**Participant characteristics**		
	***n***	**%**
Total subjects	40	100
Male gender	27	68
Northern European ethnicity	29	73
Atopic comorbidity (prior or present)	39	98
- Atopic dermatitis	35	88
- Asthma	31	78
- Rhinoconjunctivitis	27	68
Atopic disposition (1st degree relative)	33	83
		Median age in years (range)
Age at inclusion		8 (2–23)
IgE in kU/L (whole nut extract)	*n* subjects with sIgE	sIgE all subjects, median (range)
- Hazelnut (f17)	33	11.9 (<0.35–141)
- Birch pollen (t3)	22	0.85 (<0.35–405)
- Walnut (f256)	33	4.1 (<0.35–95.7)
- Pistachio nut (f203)	37	5.6 (<0.35–87.9)
- Cashew nut (f202)	33	4.4 (<0.35–167)
- Almond (f20)	22	0.43 (<0.35–37.3)
- Peanut (f13)	29	1.7 (<0.35–457)

*Northern European ethnicity is used as a term for patients where both parents are of Northern European ancestry (predominantly Danish)*.

### Allergic Phenotypes and IgE Serology

All 40 participants were assessed for clinically relevant allergy to at least one tree nut.

**Figure 1:**
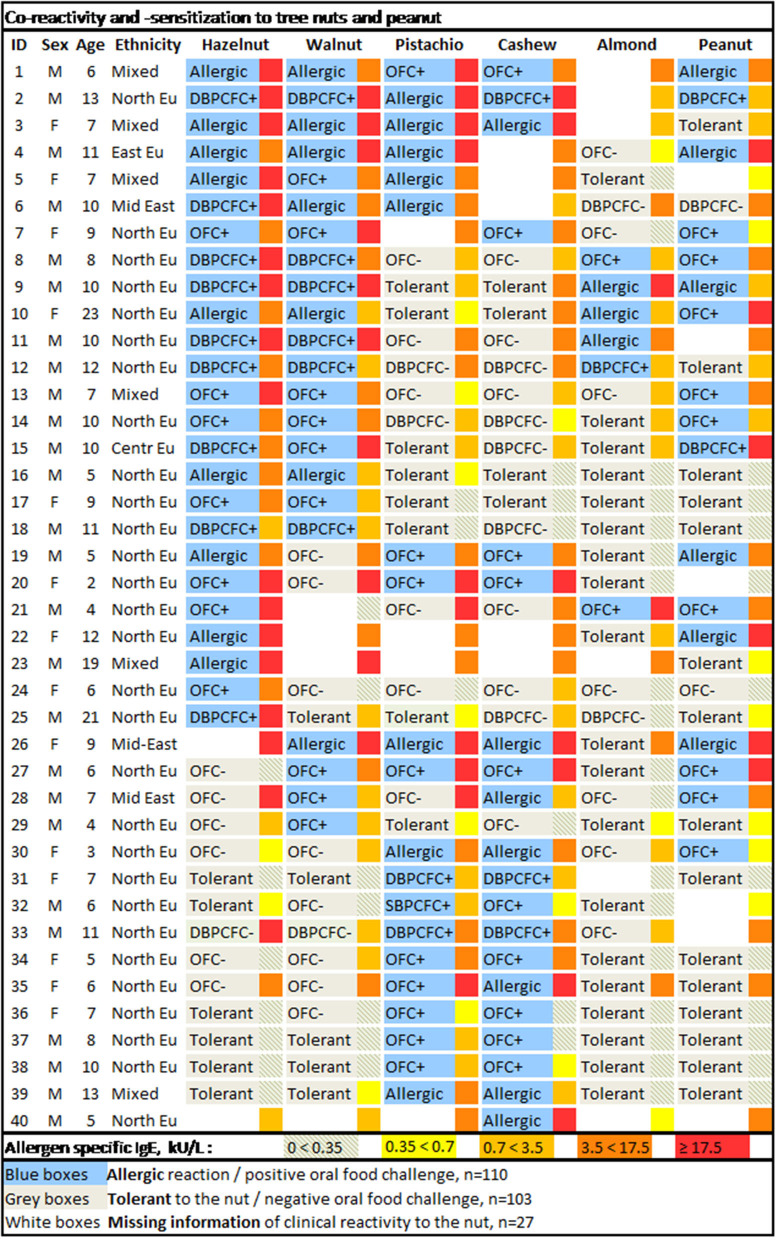
Co-reactivity and -sensitization to tree nuts and peanut. The allergen-specific IgE levels are depicted in the narrow box adjacent to the right of the broader boxes withholding information on clinical reactivity to the specific nut. M, male; F, female; Ethnicity: North EU, Northern European (predominantly Danish); East EU, Eastern European; Mid East, Middle Eastern; Centr Eu, Central European; Mixed, Northern European and another ethnic ancestry (predominantly African).

Thirty-one participants underwent a total of 116 OFCs (excluding placebo challenges). For the remaining, the diagnosis relied on a convincing medical history of either allergy or tolerance. Twenty-three participants were assessed for allergy to all six nuts. For the remaining 17 participants, there was missing information of clinical reactivity in 27 patient-food instances. Patterns of co-reactivity toward the tree nuts and peanut along with a heat map of sIgE levels are illustrated in Figure 1.

Allergy to hazelnut, walnut, pistachio, cashew, almond, and peanut was confirmed in 25, 22, 20, 20, 6, and 17 participants, respectively. There were no placebo reactions in the DBPCFCs (data not shown). Allergy was diagnosed to other nuts (not systematically assessed) and found to macadamia in one subject and allergy to pecan in three participants (all of whom were also allergic to walnut) (data not shown). No participants had confirmed allergy to Brazil nut.

Most participants had confirmed allergy to at least two nuts (35 subjects, 86%), over half (22 subjects, 55%) had confirmed allergy to three nuts, and 11 participants (28%) were confirmed allergic to four nuts. Only two out of all 40 participants had confirmed allergy to five nuts (hazelnut, walnut, pistachio, cashew, and peanut). None had confirmed allergy to all six nuts, although due to missing data, three subjects could potentially be allergic to all nuts.

Regarding tree nuts, 34, 16, and 3 participants were confirmed to react to at least two, three, or four tree nuts. In 21 instances (distributed over 13 participants), the reactivity against one or more tree nuts could not be established. Allergy to tree nuts could therefore potentially be as high as 37 subjects with allergy to 2+ tree nuts, 21 with allergy to 3+ tree nuts, 11 with allergy to 4+ tree nuts, and 4 subjects with potential allergy to all 5 tree nuts.

There were two independent major allergic phenotypes: dual allergy to the pairs hazelnut-walnut and pistachio-cashew nut (Figure 1). At least 18 out of the 40 participants (45%) were dual allergic to hazelnut and walnut, and 10 subjects (25%) were tolerant to both. Seventeen out of all 40 participants (43%) were dual allergic to pistachio nut and cashew nut, and 15 subjects (38%) were tolerant to both nuts. There were 33 subjects that were assessed for clinical reactivity to both cashew and pistachio nut, out of which only one of the 18 cashew nut allergic subjects tolerated pistachio (3%). There were no pistachio nut allergic subjects that tolerated cashew nuts.

Among our selected tree nuts, almonds turned out to be the least allergenic, as only six out of the 40 participants (15%) were confirmed allergic to almonds. Out of those with confirmed almond allergy, none was allergic to cashew nut and pistachio nut, five were allergic to both hazelnut and walnut, and three were allergic to hazelnut, walnut, and peanut.

Seventeen participants (43%) had confirmed allergy to peanuts. However, the number of tree nut allergic subjects with co-allergy to peanut could potentially be as high as 23 (56%), as there were missing data on clinical reactivity to peanut in six instances.

Interestingly, no distinct pattern of tree nut reactivity could be found in those patients that also reacted to peanuts.

### Symptomology, Rescue Medication, and Threshold Dose in OFCs

There were a total of 70 positive OFCs (Table 3). Most positive challenges resulted in symptoms from two or more organ systems. The most common symptoms were oral symptoms (79%) followed by skin involvement (70%). Almost half of the subjects with positive OFCs had gastrointestinal symptoms (46%) with overweight in subjects challenged with pistachio nut (58%) and cashew nut (77%). Symptoms in upper airways were seen in a little less than half the cases (44%) and lower respiratory symptoms in a quarter of subjects (26%). Behavioral change was reported in 13% but was mostly observed in younger children (data not shown). No subjects had cardiovascular or neurologic symptoms.

**Table 3:** Positive oral food challenges: clinical features and medical treatment.

**Positive oral food challenges: clinical features and medical treatment**							
**Positive challenges**	**Hazelnut *n* = 16**	**Walnut *n* = 15**	**Pistachio *n* = 12**	**Cashew *n* = 13**	**Almond *n* = 3**	**Peanut *n* = 11**	**Total *n* = 70**
**Symptoms (%)**							
**Oral symptoms** Itchy mouth, throat and ears, clearing throat	12 (75)	12 (80)	12 (100)	10 (80)	2 (67)	7 (64)	55 (79)
**Skin involvement** Angioedema, pruritus, rash, flare up of eczema, flushing, urticaria	11 (69)	14 (93)	8 (67)	10 (78)	1 (33)	5 (45)	49 (70)
**Gastrointestinal symptoms** Nausea, stomach ache, diarrhea, vomiting	4 (25)	5 (33)	7 (58)	10 (77)	1 (33)	5 (45)	32 (46)
**Upper airways** Rhinitis, conjunctivitis, sneezing	8 (50)	8 (53)	4 (33)	7 (54)	1 (33)	3 (27)	31 (44)
**Respiratory symptoms** Wheeze, rhonchi, stridor, cough, hoarseness	5 (31)	4 (26)	2 (16)	4 (31)	0	3 (27)	18 (26)
**Behavioral change** Invertedness, agitation, discomfort, crying, weak	1 (6)	1 (7)	3 (25)	4 (31)	0	0	9 (13)
**Cardio-vascular change** Tachycardia, drop in blood pressure > 20%, collapse	0	0	0	0	0	0	0
**Neurologic symptoms** Fainting, unresponsiveness	0	0	0	0	0	0	0
**Medication required (%)**							
**None**	4 (25)	4 (27)	5 (42)	6 (46)	2 (67)	7^*^ (70)	28^*^ (41)
**Adrenaline** (intramuscular)^**^	1 (6)	2 (13)	1 (8)	0	0	0^*^	4^*^ (6)
**Beta-2 agonist** (inhalation)	0	4 (27)	2 (17)	3 (23)	0	0^*^	9^*^ (13)
**Antihistamine** (orally / intravenous)	12 (75)	11 (73)	7 (58)	7 (54)	1 (33)	3^*^ (30)	41^*^ (59)
**Corticosteroid** (orally / intravenous)	6 (38)	7 (47)	3 (25)	5 (38)	0	1^*^ (10)	22^*^ (32)

* *Missing information regarding medicine in 1/11 peanut challenges (not included in percentage calculations)*.

** *Adrenaline was administered to four different subjects*.

*Thirty-two participants had one or more positive food challenges*.

*Symptoms are reported in absolute numbers and in percent of the positive challenges to the given nut*.

*Medication required is reported in absolute numbers and in the percentage of positive challenges*.

Medication was administered in 59% of challenges out of which antihistamine was administered in all cases and corticosteroids in 32%. Beta-2 agonists were administered in 13% of challenges and in 6% of challenges (four different subjects, data not shown) received adrenaline (epinephrine; Table 3).

**Figure 2:**
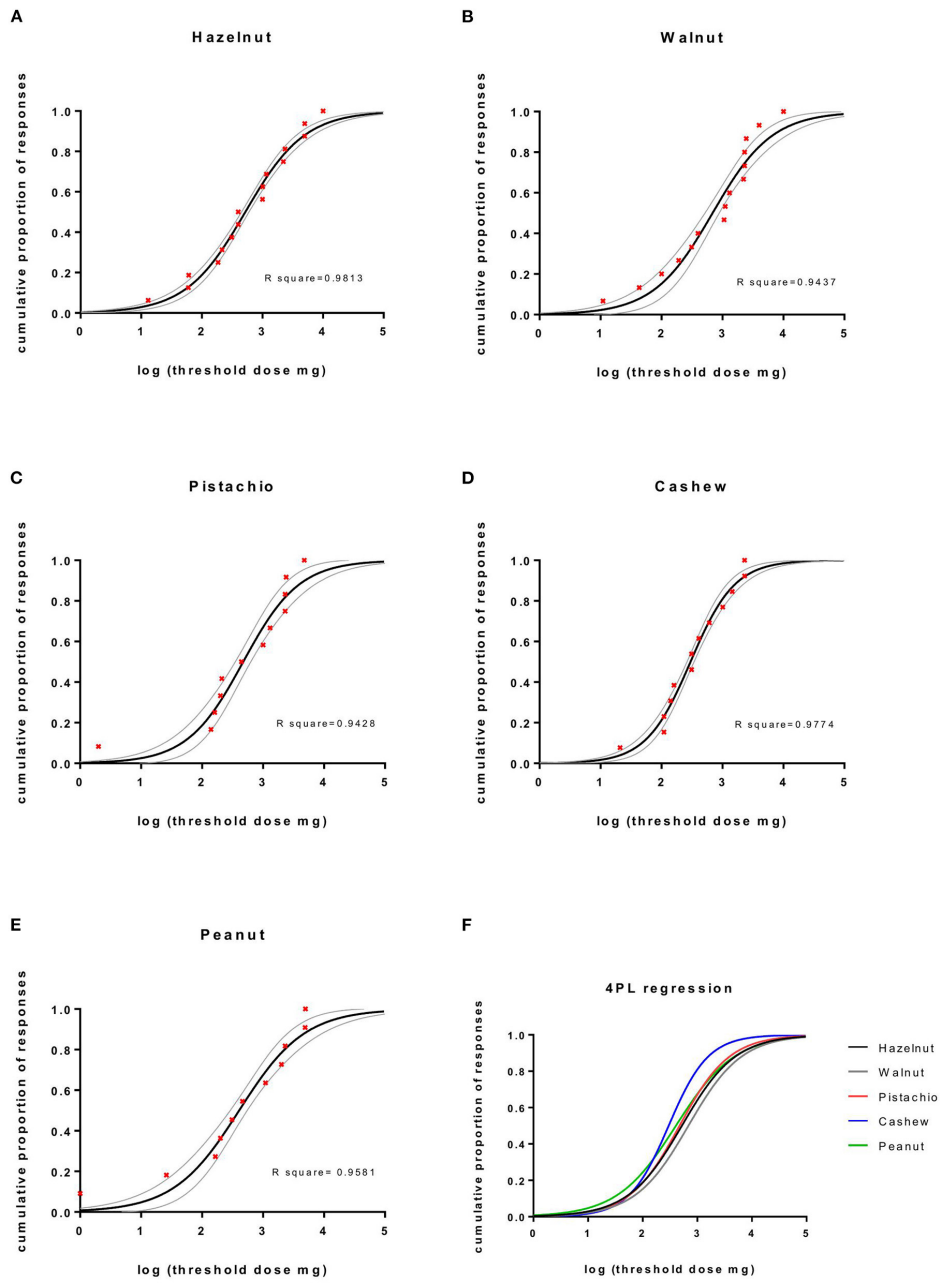
The cumulative proportion of responses against the threshold doses to five nuts. Four parametric logistic regression curves (constraints top = 1, bottom = 0). **(A–E)** The cumulative proportion of responses against log threshold doses of nuts, 95% CI and **(F)** combined dose-response curves.

The cumulative proportion of responses against the threshold doses to five nuts is shown in Figure 2. The half-maximal effective cumulative challenge dose, EC50, is an estimate for the threshold dose where 50% of the participants with positive OFCs will react. EC50 (95% confidence interval [CI]) doses were as follows: hazelnut 522 mg (454–601), walnut 688 mg (529–894), pistachio nut 473 mg (356–630), cashew nut 299 mg (262–341), and peanut 406 mg (297–554) of whole dried nut, which is equivalent to a protein content of 74 mg (60–80) in hazelnut, 98 mg (76–128) in walnut, 98 mg (73–130) in pistachio nut, 46 mg (40–52) in cashew nut, and 105 mg (77–143) in peanut. Ordinary one-way ANOVA with Bonferroni's multiple comparisons test of the combined dose-response curves slopes renders significant differences in EC50 threshold doses between hazelnut vs. cashew nut *p* = 0.002, cashew nut vs. pistachio nut *p* = 0.0308, walnut vs. cashew nut *p* < 0.0001, and walnut vs. peanut *p* = 0.008.

Since only 3 almond challenges were performed, it was not possible to produce a curve or to calculate EC50. The median and range of the cumulative threshold dose of whole dried almond was 1,000 mg (311–10,000) equivalent to 212 mg (66–2,120) of protein. Data on protein content were obtained from frida.fooddata.dk.

### Levels of SIgE

**Figure 3:**
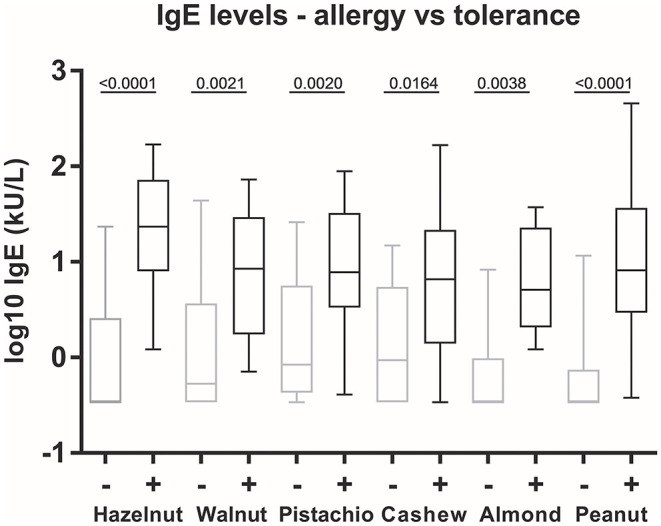
Allergen-specific IgE levels in allergy vs. tolerance. The box plot shows the median, 25 and 75% percentiles (box), and minimum and maximum (whiskers) values of log10 values of sIgE to whole nut extracts. Statistics: One-way ANOVA followed by Bonferroni's multiple comparison *post-hoc* tests. +, allergy to the nut; –, tolerance to the nut.

There were significant differences in levels of sIgE to all nuts between allergic and tolerant subjects (Figure 3). There were, however, no significant differences between sIgE levels to individual nuts in sera from subjects allergic to the nuts when performing multiple comparisons, i.e., no nut had a markedly higher or lower sIgE level than the other nuts.

All but two subjects (both cashew nut allergic assessed by food challenges) had sIgE above detection limit to all the nuts they were allergic to (heat map in Figure 1). Tolerance was predicted in the remaining 48 assessed cases with sIgE < 0.35 kU/L.

**Figure 4:**
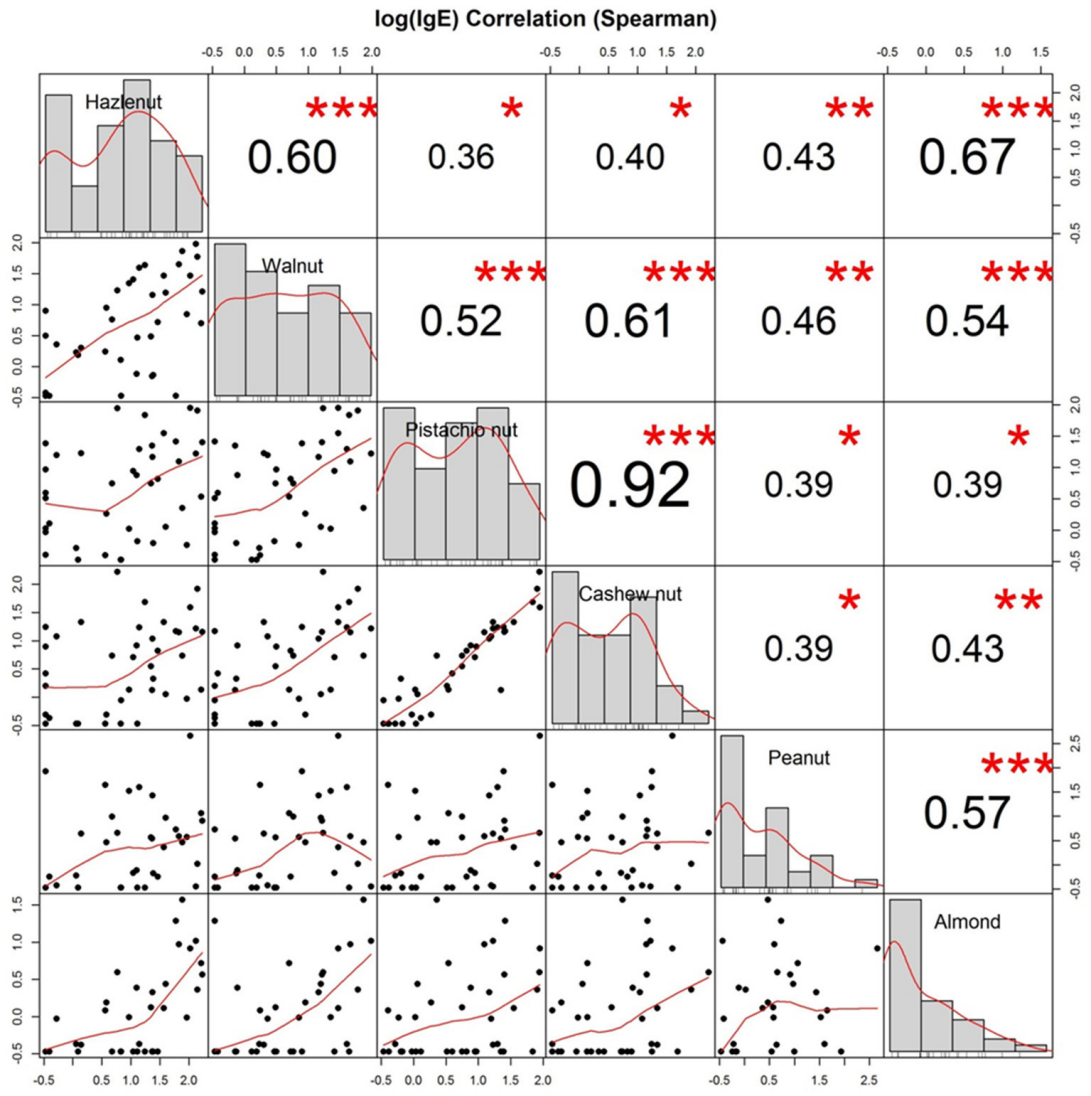
Distribution and allergen-specific IgE correlations between nuts. Distribution (diagonal), Scatterplots (bottom of diagonal), and Spearman correlation coefficients (top of diagonal) between allergen-specific IgE toward whole extracts of hazelnut, walnut, cashew nut, pistachio nut, peanut, and almond from all 40 participants. Logarithmic values of IgE are shown. Stars indicate level of significance: *p* (<0.001, <0.01, and <0.05) <, =, > symbols (***, **, *).

Allergen-specific IgE distributions and scatter plots of sIgE to each pair of nuts are depicted in Figure 4. Spearman's rank-order correlation coefficients (r_s_) were calculated for sIgE to each pair of nuts. There was a very strong correlation between the level of sIgE to cashew nut and pistachio nut (0.92) and a strong correlation between sIgE to hazelnut and almond (0.67), walnut and cashew nut (0.61), and hazelnut and walnut (0.60).

## Discussion

This is a comprehensive study of clinical co-reactivity to multiple nuts in a Northern European study population. The study has systematically investigated sIgE levels, clinical reactivity, and established threshold levels for clinical reactivity to commonly ingested tree nuts and peanuts. The study participants all underwent thorough medical assessment, and the clinical diagnosis was based on food challenges whenever the clinical reactivity was unestablished or uncertain. The evaluation of allergy vs. tolerance to five tree nuts together with peanuts in the same group of patients all with clinically relevant tree nut allergy makes this study distinctive.

The study demonstrated that subjects with a clinically relevant tree nut allergy were allergic to two nuts in most cases and often allergic to three tree nuts. Hazelnut-walnut dual allergy was common. Allergy to cashew nut was in all but one case observed with a concomitant allergy to pistachio nut, reflected by a very strong sIgE correlation between the nuts. Half of all the assessed subjects were allergic to peanuts as well.

Symptoms from the oral cavity followed by a skin reaction were the most common sequence in positive OFCs, and subjects often presented with symptoms from two or more organ systems. The dose-response curves for food challenges performed with whole nuts were similar in most cases, although significant differences in EC50 threshold doses were found between hazelnut vs. cashew, cashew vs. pistachio, walnut vs. cashew, and walnut vs. peanut. Cashew nut tended to cause allergic symptoms at the lowest dose when compared to the other tree nuts.

The majority of the tree nut and especially hazelnut sensitization seen in Northern Europe is secondary to birch pollen sensitization often causing no symptoms at all or subjective or milder symptoms (13, 33). We wanted a representative group of tree nut allergic individuals, where we would expect a high degree of atopic co-morbidity (34) that includes birch pollen sensitization characteristic of the Scandinavian area. We found no significant differences in sIgE to the tree nuts and birch pollen in the patients who were recruited through the database search as compared with the newly referred patients, indicating that the study population is representative of most patients referred with a clinically relevant tree nut allergy in the capital region of Denmark.

The participants were all diagnosed with allergies to at least one tree nut upon entry. One could speculate that patients with multiple and more severe allergies were more likely to be referred to the clinics, resulting in a recruitment bias. There are no current data on the rate of tree nut allergy in the Danish background population. We did not perform a mitigation analysis of our population to the general Danish population. However, our study findings, that tree nut allergic individuals in a Northern European population are often allergic to more than one tree nut, are comparable to findings based on a large birth cohort in Stockholm (BAMSE cohort, Karolinska Institutet) (35).

We avoided using component resolved diagnostics (CRD) in our search criteria. Probably not all relevant components in tree nuts are commercially available at present and the association between sensitization and allergic symptoms has not been fully revealed (36). Using CRD for selecting a tree nut allergic population could potentially leave out patients with clinically relevant allergies to perhaps not yet known components. If we had used CRD in our inclusion process, we may possibly have missed some lesser common patterns of sensitization that accounts for clinical reactivity. A study that characterizes the present cohort's allergen component profile is underway.

A limitation of the study was that there were no teens aged 14–18 at the time of inclusion, although we aimed to include all eligible patients. The difficulty in recruiting older patients was due to their busy schedule and many stated that they did not want further food challenges.

It would have been optimal to expand the study to include more tree nuts, but the timeframe of the study did not allow that since food challenges are both time- and resource-consuming to perform. Therefore, we chose to investigate the nuts most commonly ingested in the Danish population and/or reported as offenders. A feasibility study demonstrated that hazelnut, walnut, cashew nut, pistachio nut, almond, and peanut were the most common nuts causing allergic reactions (data not shown).

Oral food challenges (OFCs) are considered the gold standard when dealing with food allergy (37), but challenges do not always provide definite or stationary results. Some patients may have been tested negative in an OFC, but then react to the nut on subsequent exposure (38) or under different conditions (cofactors or concomitant disease) (39). The timing and order of challenges to different nuts could possibly affect the outcome and new allergies may develop over time depending on the age of the patient (34, 40). We decided to include historic data on OFCs performed prior to enrolment along with a convincing medical history of either allergy or tolerance to save time and to avoid redundant exposure in case of unambiguous reactions.

We set out to investigate patterns in clinical reactivity to tree nuts and peanuts. It has not been established whether sensitization toward multiple nuts is mainly due to co- or cross-sensitization (5). Clinical co-allergy to hazelnuts and peanuts is found to be associated with cross-reactive T-cell responses (41, 42), and in a mouse study, immunotherapy with a single tree nut (cashew) also attenuated walnut and pistachio allergy (43). In line with recent findings in an inhibition study (44), one could therefore speculate whether there are one or two sensitizer nuts that primarily drive the development of allergy to the other nuts.

Even though allergy to two or more nuts was common in this cohort, a clear differentiation between allergic phenotypes was observed; subjects that were allergic to hazelnuts and walnuts but tolerant to pistachio nuts and cashew nuts or vice versa. Only a few participants were allergic to all the tree nuts tested for. The division in phenotypes is also seen in a large Scandinavian study on sensitization profiles (13), suggesting that the patterns of clinical reactivity are caused by cross-sensitivity between taxonomically related foods.

Dual allergy to cashew nuts and pistachio nuts was seen in all but one participant. This dual allergy was also found in a Greek study, where all of 25 children with a definite diagnosis reacted to both nuts (45). In the present study, we found that dual allergy to hazelnut and walnut was also common, but almost a third of the participants with allergy to one of the nuts were tolerant to the other. All of the participants diagnosed with almond allergy were allergic to hazelnuts, and all almond allergic participants that were assessed were also allergic to walnut. All of the assessed subjects that reacted to almonds were tolerant to cashew nut and pistachio nut. This finding differed from a study out of Stanford University, USA by Andorf et al. (26), where co-allergy to almonds, cashew, and pistachio was more common. In a study by Elizur et al. (27) assessing co-reactivity between six tree nuts in 83 tree nut allergic individuals in an Israeli population, almond allergy was only found in one case. The differences in clinical reactivity between the present and the abovementioned studies are likely due to geographic differences in exposure to allergens and to cross-reacting allergens.

A newer multicenter study by Brough et al. has in line with our findings found strong clinical correlations between cashew and pistachio and between walnut and hazelnut (9). The study however also found regional differences in allergy profiles.

At least 43% of the participants in this study were allergic to peanut. The high numbers of concomitant peanut and tree nut allergy were also found in a Northern American study based on questionnaires where 34% of tree nut allergic individuals also reported peanut allergy (46). In the present study allergy to peanuts was not confined to a single phenotype, but it was often present in participants with a concomitant allergy to hazelnut, walnut, and almond, and often present in cases where participants were allergic to three or four other nuts.

We observed possible correlations with gender and age that could be linked to the patterns of clinical reactivity. Subjects that were eligible for inclusion in the present study were predominantly of the male gender. The larger male representation in the younger age group of individuals with a food allergy does not differ from other cohort studies (47, 48). Moreover, our finding of a high rate of atopic co-morbidity was also in line with prior study findings (7, 35).

The patterns in clinical reactivity are reflected by correlations between sIgE to whole nut extracts. We saw a very strong correlation between sIgE to cashew nuts and pistachio nuts, in line with findings in the Greek study with a near to perfect correlation (r_s_ = 0.98) (45). Moreover, in agreement with a diagnostic study of predicting positive food challenges (25), we found that the prevalence of clinical reactivity was increased with increasing sIgE levels, although clinical reactivity was also present at low levels of sIgE and even at sIgE below the detection limit.

The most common reactions observed during positive OFCs were subjective symptoms from the oral cavity followed by objective symptoms from the skin. This combination of symptoms is a cessation criterion in line with the general understanding of when to stop an OFC (31). Rescue medication administered in OFCs reflects the severity of the clinical reaction, but the use is influenced by the patients' comorbidity and prior allergic reactions to other foods (49). Factors, such as a high cumulative threshold dose or food retention caused by nausea (50), which could lead to prolonged allergen exposure, could also have affected the readiness to administer rescue medication and thereby pose a bias.

The lowest EC50 dose of allergen was observed for reactions to cashew nuts. Other studies have likewise reported that the cashew nut allergic patients react to minimal amounts of nut (51, 52) and often experience more severe reactions compared to other nut allergies (53), reflecting the allergenic potency of the nut protein.

This study has demonstrated that single tree nut allergy is rare in this cohort of Danish patients with a clinically relevant tree nut allergy. Co- or cross-reactivity should therefore always be evaluated in a tree nut allergic individual. The results may be influenced by cross-sensitization with pollen or other plant foods, but the results still provide insight on the pattern of tree nut allergies in a Northern European pediatric and young adult population. A planned follow-up study of the clinical reactivity in relation to the presence of IgE to specific allergen components will give further insight into the level of the observed co- or cross-reactivity. Immunological studies of antibody-facilitated allergen presentation (54) and T-cell epitope cross-reactivity (41) could provide plausible connections to the clinical patterns in tree nut allergy that we have observed. Our clinical knowledge combined with *in silico* and *ex vivo* approaches may ultimately propose a pathogenesis model of the development of multiple tree nut and plant food allergies, which is essential knowledge regarding new or improved diagnosis, therapy, and finally prevention.

## Data Availability Statement

The raw data supporting the conclusions of this article will be made available by the authors, without undue reservation.

## Ethics Statement

The studies involving human participants were reviewed and approved by the local Danish Ethical committee, ID: H-6-2014-018. All participants (or their legal guardians) signed informed consent for participation. Written informed consent to participate in this study was provided by the participants' legal guardian/next of kin.

## Author Contributions

NJ-B, KH, and LP contributed to the conception and design of the study. NJ-B and LL performed the statistical analysis. NJ-B organized the database and wrote the first draft of the manuscript. NJ-B, NK, IN, and KH conducted participant enrolment and performed diagnostic tests. All authors contributed to manuscript revision, read, and approved the submitted version.

## Funding

The study received a grant from Grethe Stampe's scholarship for research in food allergy and allergic lung disorders, c/o Astma-Allergi Danmark, Roskilde, Denmark. No other funding was received.

## Conflict of Interest

The authors declare that the research was conducted in the absence of any commercial or financial relationships that could be construed as a potential conflict of interest.

## Publisher's Note

All claims expressed in this article are solely those of the authors and do not necessarily represent those of their affiliated organizations, or those of the publisher, the editors and the reviewers. Any product that may be evaluated in this article, or claim that may be made by its manufacturer, is not guaranteed or endorsed by the publisher.
